# ICTV Virus Taxonomy Profile: Chaseviridae 2022

**DOI:** 10.1099/jgv.0.001715

**Published:** 2022-04-13

**Authors:** Hany Anany, Padmanabhan Mahadevan, Dann Turner, Evelien M. Adriaenssens, Andrew M. Kropinski, ICTV Report Consortium

**Affiliations:** 1 Guelph Research and Development Centre, Agriculture and AgriFood Canada, Guelph Ontario N1G 5C9, Canada; 2 Department of Biology, The University of Tampa, Tampa, FL 33606-1490, USA; 3 Department of Applied Sciences, University of the West of England, Bristol Frenchay Campus, Bristol BS16 1QY, UK; 4 Quadram Institute Bioscience, Norwich Research Park, Norwich NR4 7UQ, UK; 5 Department of Pathobiology, University of Guelph, Guelph, Ontario N1G 2W1, Canada

**Keywords:** ICTV Report, taxonomy, *Chaseviridae*

## Abstract

Members of the family *Chaseviridae* are lytic bacterial viruses infecting representatives of the bacterial class Gammaproteobacteria. Chaseviruses have a global distribution. Virions of members of this family have a myovirus morphology (icosahedral head with contractile tail). Genomes are dsDNA of 52–56 kbp with G+C content ranging from 39.3–52.5 %. Chaseviruses, like members of the family *Autographiviridae*, encode a large single subunit RNA polymerase, but unlike those viruses their promoter sequences have not yet been identified. This is a summary of the International Committee on Taxonomy of Viruses (ICTV) Report on the family *Chaseviridae,* which is available at ictv.global/report/chaseviridae.

## Virion

**Fig. 1.**
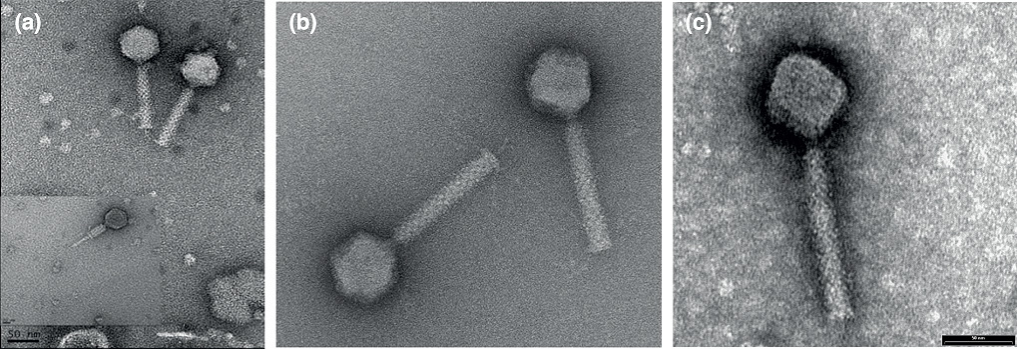
Transmission electron micrographs of negatively stained phages. (**a**) Escherichia phage vB_EcoM-4HA13, (**b**) Erwinia phage vB_EamM-Y2 [2] (provided by Martin J. Loessner), (c) Escherichia phage phiEcoM-GJ1 [3,4] (image provided by Nidham Jamalludeen).

 Virions have isometric, icosahedral heads with a diameter of 53–65 nm. The heads show clear capsomers, i.e. the subunits of the capsid are arranged in pentons and hexons that are assembled into the isometric, icosahedral capsid. The contracted tails are 116–166 nm in length. (Table 1, Fig. 1).

**Table 1.** Characteristics of members of the family *Chaseviridae*

**Example:**	Escherichia phage vB_EcoM-4HA13 (MN136198), species *Sabourvirus sv4HA13*, genus *Sabourvirus*
Virion	Head–tail morphology with contractile tail; heads generally isometric with diameters of 53–65 nm showing capsomers; uncontracted tails 116–166 nm in length
Genome	Linear, terminally redundant, non-permuted dsDNA of 52–56 kbp
Replication	Phage-encoded DNA polymerase
Translation	Bacterial translation
Host range	Bacteria of the phylum Proteobacteria, class Gammaproteobacteria
Taxonomy	Realm *Duplodnaviria*, kingdom *Heunggongvirae*, phylum *Uroviricota*, class *Caudoviricetes*; two subfamilies (*Cleopatravirinae* and *Nefertitivirinae*) with multiple genera and multiple species

## Genome

**Fig. 2.**
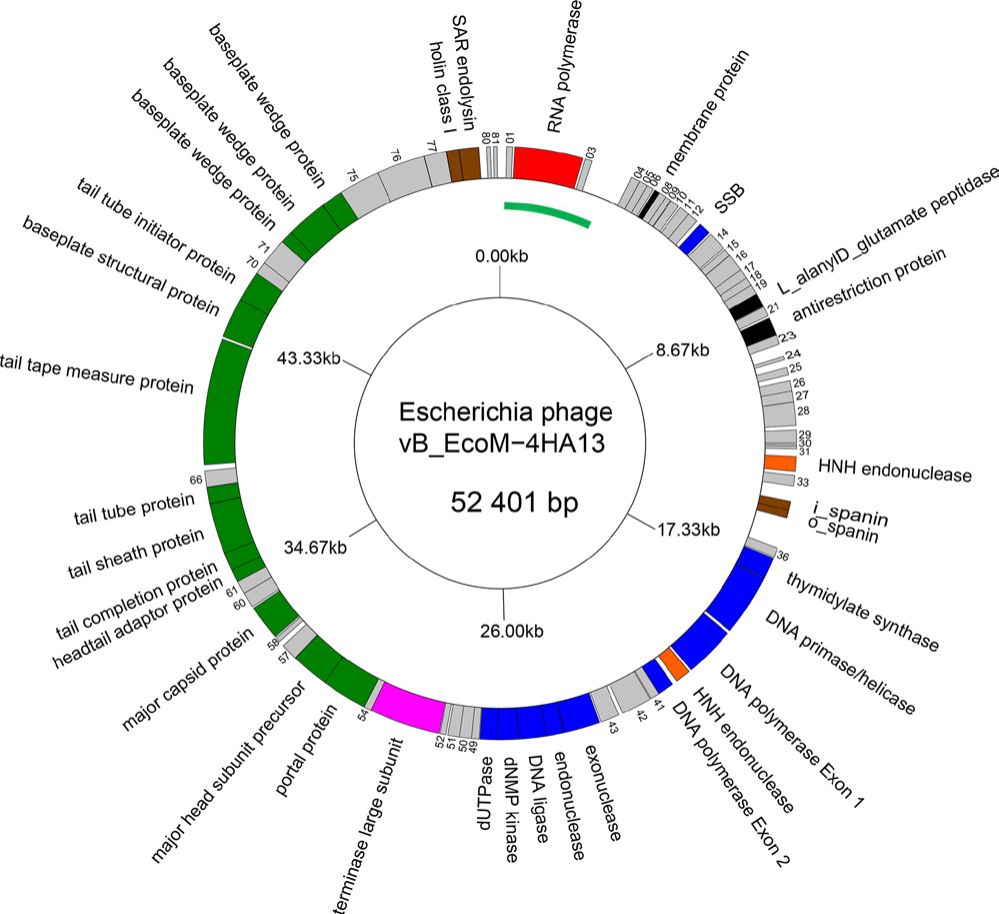
Genome organization of Escherichia phage vB_EcoM-HA13. Genes involved in transcription are coloured red; DNA and nucleotide synthesis, blue; homing endonucleases, orange; packaging, pink; morphogenesis, green; lysis, brown. The linear genome is shown as a circle with the green arc representing the left long terminal repeat. This figure was generated with GenomeVx (http://wolfe.ucd.ie/GenomeVx/ [5]) and edited with Foxit PDF Editor.

The genomes of members of the family *Chaseviridae* consist of linear dsDNA with long terminal repeats of approximately 3 kbp (Fig. 2). Genomes range between 52–56 kbp. Some members are predicted to encode tRNAs. There is no report of modified bases in the DNA of these viruses.

## Replication

Transcription is mediated by the host machinery and a virus-encoded RNA polymerase, related to that of members of the family *Autographiviridae* [1]. DNA replication is mediated by a virus-encoded DNA polymerase and DNA helicase/primase.

## Taxonomy

Current taxonomy: ictv.global/taxonomy. The family *Chaseviridae* includes two subfamilies, each with multiple genera. The subfamilies and genera are identified as well-supported monophyletic groups based on phylogenetic analysis of concatenated core gene markers and single core genes. Members of the same virus genus generally infect members of the same bacterial genus. Members of the same species are >95 % identical in nucleotide sequence over the length of the genome, including the terminal repeat region.

## Resources

Full ICTV Report on the family *Chaseviridae*: ictv.global/report/chaseviridae.
